# Direct and indirect alcohol biomarkers data collected in hair samples - multivariate data analysis and likelihood ratio interpretation perspectives

**DOI:** 10.1016/j.dib.2017.03.026

**Published:** 2017-03-16

**Authors:** Eugenio Alladio, Agnieszka Martyna, Alberto Salomone, Valentina Pirro, Marco Vincenti, Grzegorz Zadora

**Affiliations:** a Dipartimento di Chimica, Università degli Studi di Torino, Via P. Giuria 7, 10125 Torino, Italy; b Centro Regionale Antidoping e di Tossicologia “A. Bertinaria”, Regione Gonzole 10/1, 10043 Orbassano, Torino, Italy; c Department of Analytical Chemistry, Chemometric Research Group, Institute of Chemistry, The University of Silesia, Szkolna 9, 40-006 Katowice, Poland; d Department of Chemistry, Purdue University, 560 Oval Drive, West Lafayette, 47907 Indiana, USA; e Institute of Forensic Research, Westerplatte 9, 31-033 Krakow, Poland

**Keywords:** Alcohol, Likelihood ratio, Multivariate data analysis, Empirical cross entropy, Ethyl glucuronide, Fatty Acid Ethyl Esters, Hair analysis

## Abstract

The concentration values of direct and indirect biomarkers of ethanol consumption were detected in blood (indirect) or hair (direct) samples from a pool of 125 individuals classified as either chronic (i.e. positive) and non-chronic (i.e. negative) alcohol drinkers. These experimental values formed the dataset under examination (Table 1). Indirect biomarkers included: aspartate transferase (AST), alanine transferase (ALT), gamma-glutamyl transferase (GGT), mean corpuscular volume of the erythrocytes (MCV), carbohydrate-deficient-transferrin (CDT). The following direct biomarkers were also detected in hair: ethyl myristate (E14:0), ethyl palmitate (E16:0), ethyl stearate (E18:1), ethyl oleate (E18:0), the sum of their four concentrations (FAEEs, i.e. Fatty Acid Ethyl Esters) and ethyl glucuronide (EtG; pg/mg). Body mass index (BMI) was also collected as a potential influencing factor. Likelihood ratio (LR) approaches have been used to provide predictive models for the diagnosis of alcohol abuse, based on different combinations of direct and indirect alcohol biomarkers, as described in “Evaluation of direct and indirect ethanol biomarkers using a likelihood ratio approach to identify chronic alcohol abusers for forensic purposes” (E. Alladio, A. Martyna, A. Salomone, V. Pirro, M. Vincenti, G. Zadora, 2017) [1].

**Specifications Table**

**Table:** 

Subject area	*Chemistry*
More specific subject area	*Biomarkers of ethanol consumption in biological samples*
Type of data	*Tables, figures*
How data was acquired	*Analysis by Likelihood Ratio (LR) approach regarding the collected concentration values of the direct and indirect biomarkers of alcohol consumption.*
Data format	*Analyzed*
Experimental factors	*Correct classification rates and Empirical Cross Entropy (ECE) plots*[2], [3]*were employed to evaluate LR models*
Experimental features	*AST, ALT and GGT were measured by means of colorimetric assays, MCV was measured with an on-purpose hematological auto-analyzer, %CDT was determined by an ad hoc High Performance Liquid Chromatography (HPLC) reagent kit, FAEEs were detected by HS-SPME-GC/MS analysis and EtG concentrations were monitored by Ultra High Performance Liquid Chromatography - Tandem Mass Spectrometry (UHPLC–MS/MS).*
Data source location	*Centro Regionale Antidoping e di Tossicologia “A. Bertinaria”, Regione Gonzole 10/1, 10043 Orbassano, Torino, Italy.*
Data accessibility	*Data are included in this paper*

**Value of the data**

• The data reported here represent a valuable collection of all the common biomarkers of alcohol abuse used worldwide; the distinct populations of chronic and non-chronic alcohol consumers can possibly be used by other researcher to develop further interpretation models.
• The Empirical Cross Entropy plots provide a novel way to look at the effectiveness of alcohol biomarkers that other researcher may use for comparison with more traditional data representations.
• The detailed data report allows a clear comparison between univariate, multivariate and Bayesian approaches, where the latter is suggested as a benchmark for further developments.
• The mathematical background reported in the “materials and methods” section allows other researcher to transpose the offered approach to different applications.

## 1 Data

Data relative to the population of 125 individuals monitored, previously classified as either chronic (i.e. positive) and non-chronic (i.e. negative) alcohol drinker, are available in Table 1. Analysis of likelihood ratio models and its performance metrics, such as Empirical Cross Entropy plots (ECE), allowed to compare the predictive capabilities of direct and indirect biomarkers of ethanol consumption, as described in [1].

**Table 1:** Data matrix (125×12) containing the concentration values of the reference populations (i.e. individuals labeled as negative or positive) for the following target analytes: the sum of ethyl myristate, ethyl palmitate, ethyl stearate and ethyl oleate concentrations (FAEEs; ng/mg), ethyl glucuronide (EtG; pg/mg), aspartate transferase (AST, IUL^−1^), alanine transferase (ALT; IUL^−1^), gamma-glutamyl transferase (GGT; IUL^−1^), mean corpuscular volume of the erythrocytes (MCV; fL), carbohydrate-deficient-transferrin (CDT; %) and body mass index (BMI).

**Subject**	**Class**	**FAEEs**	**EtG**	**AST**	**ALT**	**GGT**	**MCV**	**CDT**	**BMI**
1	Negative	0.24	24	21	37	39	97.5	1.1	27
2	Negative	0.03	22	24	27	42	93.6	1.1	19
3	Negative	0.34	18	24	20	16	91.4	2.6	27
4	Negative	0.23	11	28	18	34	97.3	1.0	23
5	Negative	0.10	18	20	22	14	87.4	0.7	22
6	Negative	0.07	19	24	31	26	96.4	1.3	26
7	Negative	0.02	17	19	19	17	95.2	1.1	27
8	Negative	0.00	18	18	13	19	92.8	1.2	29
9	Negative	0.16	11	16	22	16	90.6	0.9	26
10	Negative	0.29	15	30	25	102	95.5	0.7	22
11	Negative	0.17	23	19	32	30	91.7	1.9	20
12	Negative	0.34	20	18	15	25	86.9	1.1	17
13	Negative	0.23	14	25	22	16	87.6	1.3	25
14	Negative	0.24	24	29	33	26	91.8	1.4	21
15	Negative	0.27	20	25	39	34	95.6	1.1	28
16	Negative	0.30	15	24	20	27	98.4	1.2	20
17	Negative	0.19	11	20	17	13	87.2	0.9	27
18	Negative	0.14	12	18	18	18	73.7	1.0	23
19	Negative	0.19	15	23	20	38	87.4	1.1	24
20	Negative	0.34	12	20	27	42	88.0	1.0	23
21	Negative	0.09	21	26	24	28	87.3	0.9	25
22	Negative	0.13	14	25	23	17	88.7	0.9	24
23	Negative	0.04	14	25	17	14	95.6	0.7	25
24	Negative	0.37	13	29	32	68	94.0	1.3	23
25	Negative	0.10	12	28	21	15	72.3	1.4	22
26	Negative	0.07	18	18	16	21	96.4	1.2	27
27	Negative	0.11	21	23	21	19	90.2	1.2	24
28	Negative	0.19	11	33	59	21	92.0	1.1	22
29	Negative	0.19	12	26	34	19	88.9	1.0	23
30	Negative	0.20	16	37	24	129	93.4	1.1	28
31	Negative	0.36	13	22	21	16	90.1	1.3	28
32	Negative	0.36	19	19	18	21	85.1	1.2	26
33	Negative	0.36	12	22	20	13	75.6	1.8	23
34	Negative	0.40	23	26	48	25	94.2	0.9	22
35	Negative	0.44	13	25	20	17	94.6	0.9	21
36	Negative	0.05	26	22	10	11	94.9	1.0	25
37	Negative	0.02	1	39	89	35	85.6	1.4	28
38	Negative	0.38	4	35	66	119	90.1	1.1	35
39	Negative	0.00	6	19	18	26	84.7	1.1	25
40	Negative	0.38	4	31	25	91	87.6	1.2	22
41	Negative	0.32	8	41	125	20	88.9	0.8	26
42	Negative	0.09	9	28	59	66	83.1	0.9	27
43	Negative	0.00	2	33	20	17	83.7	0.8	25
44	Negative	0.26	8	23	23	25	58.8	1.0	21
45	Negative	0.00	1	24	42	11	92.6	1.2	25
46	Negative	0.21	1	23	21	19	80.0	1.1	23
47	Negative	0.10	7	35	28	29	93.1	1.1	23
48	Negative	0.11	9	22	27	20	89.1	1.3	27
49	Negative	0.02	9	51	26	19	89.5	0.6	23
50	Negative	0.31	9	22	26	32	88.6	1.1	26
51	Negative	0.12	3	32	43	87	89.7	0.9	27
52	Negative	0.03	7	26	49	57	92.7	1.4	29
53	Negative	0.07	6	25	14	28	95.3	1.0	24
54	Negative	0.07	1	26	35	25	88.9	1.0	23
55	Negative	0.21	3	24	24	30	88.1	1.2	20
56	Negative	0.04	7	23	29	51	89.4	1.1	26
57	Negative	0.02	1	25	26	37	89.9	1.1	27
58	Negative	0.03	4	20	16	14	87.8	0.9	22
59	Negative	0.21	7	18	14	16	95.3	1.2	25
60	Negative	0.31	5	22	23	15	92.7	1.1	21
61	Negative	0.42	8	35	40	21	92.7	1.0	23
62	Negative	0.00	2	18	11	16	92.1	0.7	19
63	Negative	0.22	3	20	24	33	87.5	1.2	29
64	Negative	0.06	2	24	27	28	87.5	1.0	24
65	Negative	0.09	4	32	17	15	97.8	1.1	21
66	Negative	0.26	7	25	21	14	88.9	1.1	21
67	Negative	0.05	9	30	35	17	99.1	1.0	21
68	Negative	0.09	9	20	15	18	88.2	1.6	22
69	Negative	0.01	1	49	42	82	91.7	0.6	24
70	Negative	0.02	1	24	21	20	92.0	0.9	24
71	Negative	0.10	2	18	17	23	88.4	0.9	21
72	Negative	0.16	8	22	30	43	90.1	1.1	43
73	Negative	0.37	3	31	31	21	91.1	0.8	21
74	Negative	0.04	1	26	24	13	89.8	0.9	23
75	Negative	0.13	4	27	34	45	84.9	1.1	31
76	Negative	0.25	8	32	51	33	88.8	1.3	29
77	Negative	0.10	6	31	32	55	88.5	1.2	27
78	Negative	0.15	2	23	32	33	83.9	0.9	28
79	Negative	0.01	1	27	38	43	93.1	1.5	26
80	Negative	0.25	6	20	16	11	90.3	1.2	19
81	Negative	0.14	6	24	20	24	93.1	1.1	28
82	Negative	0.35	4	17	18	16	89.6	0.9	28
83	Negative	0.16	7	19	23	21	92.0	0.7	19
84	Negative	0.16	1	42	88	116	92.6	0.9	29
85	Negative	0.15	2	23	16	29	98.9	1.7	20
86	Negative	0.02	9	32	49	30	97.6	0.8	19
87	Negative	0.25	8	27	28	18	98.0	0.8	23
88	Negative	0.00	3	18	25	25	90.1	0.9	22
89	Negative	0.12	5	25	17	15	85.5	1.0	24
90	Negative	0.01	9	33	28	23	95.7	1.0	27
91	Negative	0.13	5	30	23	15	91.9	0.8	19
92	Negative	0.20	1	22	24	34	91.1	1.5	24
93	Negative	0.19	2	24	17	21	92.5	0.7	19
94	Negative	0.02	5	50	86	32	90.8	0.9	22
95	Negative	0.09	1	34	42	22	93.9	1.3	27
96	Negative	0.02	8	33	26	20	84.8	1.1	25
97	Positive	0.52	43	23	21	45	63.4	1.7	28
98	Positive	0.92	38	27	23	16	92.7	1.0	27
99	Positive	0.57	36	37	65	78	98.8	1.3	26
100	Positive	0.93	52	31	21	20	99.1	1.5	25
101	Positive	2.05	35	29	40	135	94.0	2.0	31
102	Positive	1.22	56	29	35	102	97.1	1.0	30
103	Positive	3.19	52	40	73	41	91.6	1.4	25
104	Positive	1.56	43	39	30	17	92.0	0.9	19
105	Positive	1.30	52	17	11	18	93.7	1.5	18
106	Positive	1.35	38	41	53	39	95.5	4.8	26
107	Positive	0.51	36	19	15	12	92.0	1.2	21
108	Positive	0.51	60	28	42	35	65.3	1.8	28
109	Positive	4.50	79	27	25	25	88.7	1.4	26
110	Positive	1.42	38	23	9	24	97.2	1.0	25
111	Positive	1.37	41	22	21	26	91.1	0.9	22
112	Positive	2.98	37	21	19	22	87.1	1.6	23
113	Positive	6.44	106	25	28	23	96.8	1.1	23
114	Positive	3.17	33	21	15	14	98.4	0.8	24
115	Positive	0.98	54	26	37	67	89.8	1.2	24
116	Positive	0.57	93	22	24	27	93.6	0.9	26
117	Positive	2.25	32	27	31	14	87.0	0.8	24
118	Positive	0.69	65	25	18	42	93.8	1.1	25
119	Positive	2.45	68	19	14	20	95.0	0.9	21
120	Positive	2.10	95	65	114	97	97.7	1.3	27
121	Positive	1.25	90	26	11	23	94.5	4.2	29
122	Positive	1.03	38	52	39	202	106.5	1.0	27
123	Positive	5.84	35	28	43	160	96.8	1.4	28
124	Positive	2.04	119	25	20	58	98.5	2.0	23
125	Positive	2.02	18	19	13	21	93.0	1.9	19

## 2 Experimental design, materials and methods

Ethical approval for the study was granted by the Ethical Committee of the Azienda Ospedaliero-Universitaria San Luigi Gonzaga of Orbassano (Protocol Number 0012756). Serum activities of AST, ALT and GGT were measured by means of colorimetric assays with a Roche-Cobas Integra 800^®^ auto-analyzer (Roche Diagnostic, Basel, Switzerland). MCV was measured with an ADVIA^®^ 2120 Hematology auto-analyzer (Siemens Healthcare Diagnostic, Milan, Italy). The %CDT was determined by the HPLC reagent kit purchased by BioRad^®^ (Munich, Germany). FAEEs were detected by HS–SPME–GC/MS analysis and a MultiPurpose Sampler Flex A05-FLX-0001 (Est Analytical, West Chester Township, OH, USA) equipped with a 65 μm StableflexTM polydimethylsiloxane/divinylbenzene fiber (PDMS/DVB) from Supelco (Sigma-Aldrich, Milan, Italy) was used in combination with a 6890N GC 5975-inert MSD (Agilent Technologies, Milan, Italy). EtG concentrations were monitored by UHPLC–MS/MS analysis and a Shimadzu Nexera UHPLC system (Shimadzu, Duisburg, Germany) interfaced to an AB Sciex API 5500 triple quadrupole mass spectrometer (AB Sciex, Darmstadt, Germany) was employed.

Descriptions about the analytical methodologies utilized to detect both the direct and the indirect biomarkers are available in [1] and [4].

**Fig. 1:**
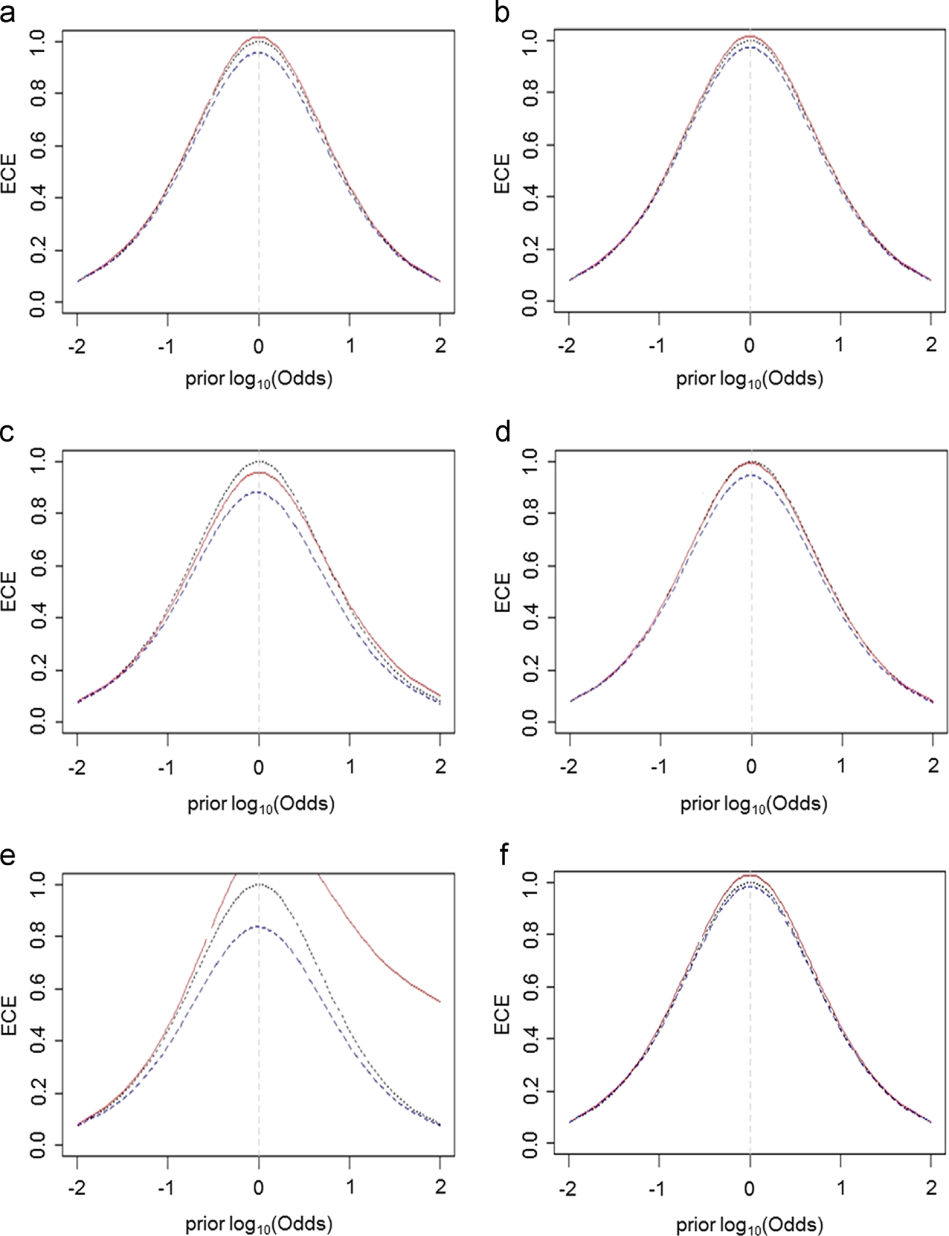
The ECE plots describing the performance of univariate LR models relative to ALT (a), AST (b), CDT (c) and GGT (d), MCV (e) and BMI (f) variables. These plots suggest that the indirect biomarkers detected in blood samples prove inadequate to provide clear discrimination between chronic from non-chronic alcohol consumers, as measured by both correct classification rates and ECE plots.

Base 10 logarithm transformation (log_10_*x*) was applied on the analyzed data. Before calculating the different LR models, all the variables were autoscaled and equal prior probabilities were utilized. LR evaluations (briefly represented by this formula LR=Pr(E|H_1_)/Pr(E|H_2_)) involved two mutually exclusive hypotheses (H_1_: the subject is not a chronic alcohol abuser – “negative” class; H_2_: the subject is a chronic alcohol abuser – “positive” class) and a reference population was used to build the model, representing the experimental evidence (E). The ECE plots relative to indirect biomarkers detected in blood samples are reported in Fig. 1.

**Fig. 2:**
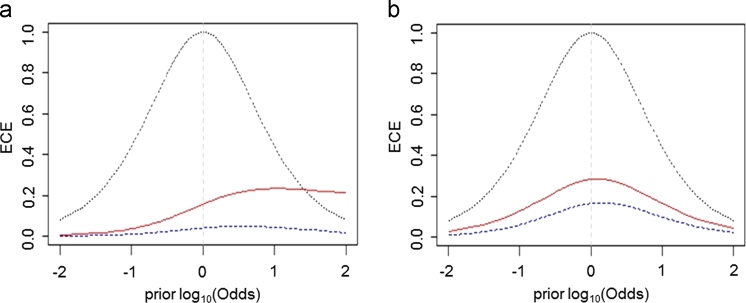
The ECE plots describing the performance of LR models relative to all the variables (LR_8_)(a) and CDT, GGT, FAEEs and EtG only (LR_4_) (b).

ECE plots relative to the sum of the four FAEEs and EtG are reported in [1]. Further LR models were tested combining biomarkers, providing higher performances. As an example, LR models developed taking into account all the variables simultaneously (LR_8_, i.e. AST, ALT, GGT, CDT, MCV, BMI, FAEEs and EtG) and a shorter list of variables (LR_4_, i.e. CDT, GGT, FAEEs and EtG) are shown in Fig. 2a–b.

**Fig. 3:**
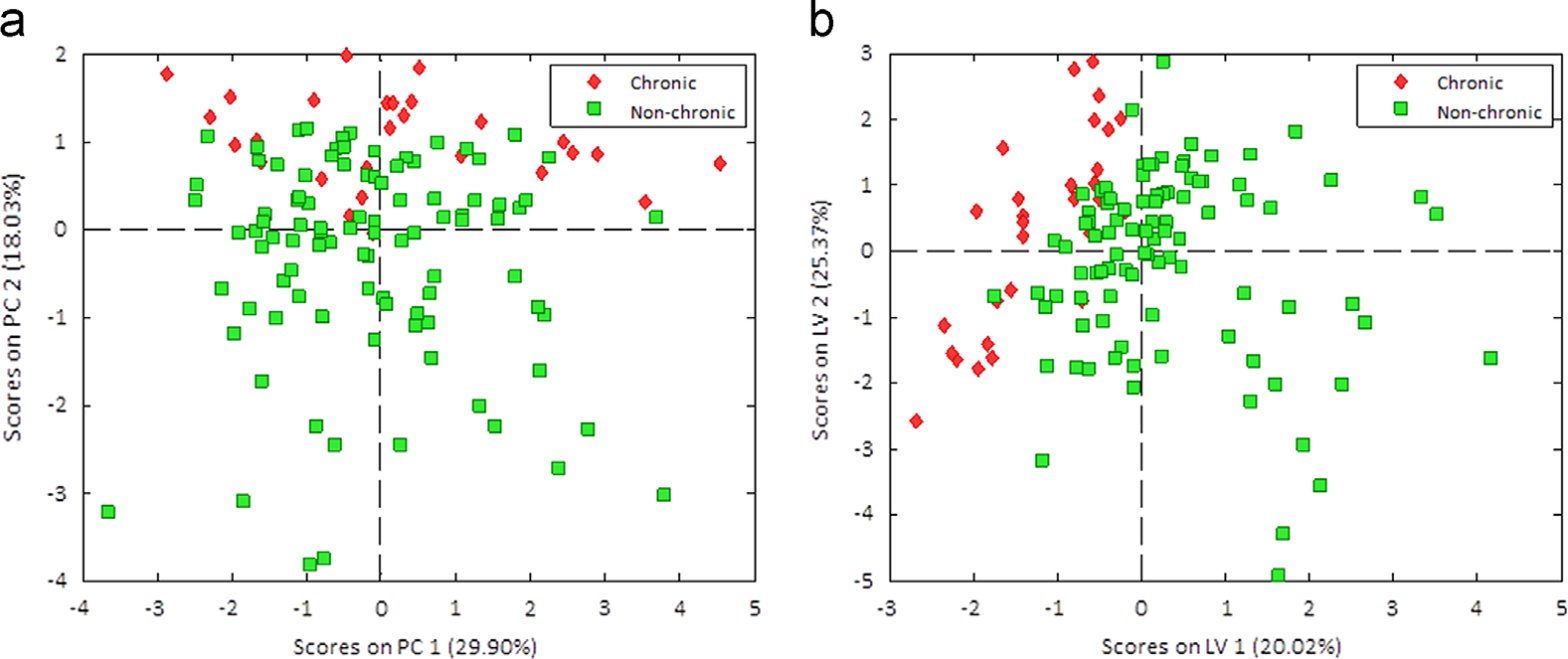
The PCA(a) and PLS-DA (b) Score Plots: chronic alcohol drinkers are represented by red diamonds, while non-chronic alcohol drinkers are indicated by green squares.

Multivariate approaches were also performed on the collected data simultaneously; Principal Components Analysis [5] (PCA, Fig. 3a) and Partial Least Squares – Discriminant Analysis [6] (PLS-DA, Fig. 3b).

The formulas employed, together with the description of ECE plots, are reported in Supplementary material.
