# Supplementary material for: Direct and indirect alcohol biomarkers data collected in hair samples - multivariate data analysis and likelihood ratio interpretation perspectives
Source: Data Brief. 2017 Mar 16;12:1–8. doi: 10.1016/j.dib.2017.03.026 (PMC5457474; doi:10.1016/j.dib.2017.03.026)
Supplement: Supplementary file 2 — Supplementary material [file mmc2.docx]

*The following formula provides the LR values for the evaluated univariate and multivariate models [2]:*

$LR=\frac{\left|h_{1}^{2}\mathbf{C}_{1}\right|^{-1/2}\frac{1}{m_{1}}\sum_{i=1}^{m_{1}} \exp\left\{-\frac{1}{2}\left(\mathbf{y}-\mathbf{x}_{1i}\right)^{T}\left(h_{1}^{2}\mathbf{C}_{1}\right)^{-1}\left(\mathbf{y}-\mathbf{x}_{1i}\right)\right\}}{\left|h_{2}^{2}\mathbf{C}_{2}\right|^{-1/2}\frac{1}{m_{2}}\sum_{i=1}^{m_{2}} \exp\left\{-\frac{1}{2}\left(\mathbf{y}-\mathbf{x}_{2i}\right)^{T}\left(h_{2}^{2}\mathbf{C}_{2}\right)^{-1}\left(\mathbf{y}-\mathbf{x}_{2i}\right)\right\}}$

*where* ***y*** *represents a vector of measurements values,* ***C****_1_ and*  ***C****_2_ stand for the between-object covariance matrix for the gth class of individuals ($g$= 1,2; i.e. “negative”, “positive”), $\mathbf{x}_{gi}$ is vector of the data relevant to the variables describing the i-th individual in the g-th class, and $h_{\begin{matrix} g \\ \end{matrix}}$is the optimal smoothing parameter of the Gaussian kernel probability density curve for the g-th class, which is equal to $h_{g}=h_{opt}=\left(\frac{4}{m_{g}(2p+1)}\right)^{\frac{1}{p+4}}$. In particular, in the case of univariate data, $p$ is equal to 1 since it represents the number of considered variables, and vectors and matrices are replaced by scalars (e.g.* ***C*** *is replaced by $c^{2}$).*

*About ECE, it must be remarked that CR values provide only information about the support of a given hypothesis according to a threshold value of LR equal to 1. As a consequence, since LR values are treated equally in case of misleading evidence, the strength of such support, in terms of magnitude of the LR values, is ignored. Since, for instance, a LR value of 1000 would be much worse than one equal to 10 in the case of true-H_2_ hypothesis, the order of magnitude of LR values should be taken into account when dealing with misleading evidence. In practice, strong support should be delivered to the correct hypotheses (i.e. LR>>1 for true-H_1_ and LR<<1 for true-H_2_) and, conversely, very weak support to the incorrect one (i.e. LR<1 for true-H_1_ and LR>1 for true-H_2_). As a consequence, ECE approach was developed* [2,4,8,9] *to weigh the strength of such support. This procedure estimates the LR models’ performance by means of a system of rewards and penalties with reference to the experimental LR values. In particular, logarithmic strictly proper scoring rules* [2,8,10] *are employed to assign penalties to the LR responses that wrongly support the incorrect hypothesis. Consequently, the higher the support for the incorrect hypothesis, the greater the penalty to be assigned to the LR model, as follows:*

- if H_1_ is true: $-\log_{2}\left(\Pr\left(H_{1}\left|E\right.\right)\right)$
- if H_2_ is true: $-\log_{2}\left(\Pr\left(H_{2}\left|E\right.\right)\right)$

*The overall penalty rate (LS) stands for the weighted average of all the penalty values assigned to the LR model’s responses, under H_1_ and H_2_ propositions (i.e. H_1_: the object belongs to the first category; H_2_: the object belongs to the second category):*

$LS=-\frac{1}{N_{1}}\sum_{i\in cat.1} \log_{2}\left(\Pr\left(H_{1}\left|e_{i}\right.\right)\right)-\frac{1}{N_{2}}\sum_{j\in cat.2} \log_{2}\left(\Pr\left(H_{2}\left|e_{j}\right.\right)\right)$

*where$N_{1}$and $N_{2}$ represent the number of objects originally belonging to each of the categories, indicated by $i$ and $j$ indices.*

*ECE is an adjustment of LS, which is weighted by the relevant prior probabilities Pr(H_1_) and Pr(H_2_). Therefore, according to Bayes’ theorem, the LR model can be evaluated by means of the following formula:*

$ECE=\frac{\Pr\left(H_{1}\right)}{N_{1}}\sum_{i\in cat.1} \log_{2}\left(1+\frac{\Pr\left(H_{2}\right)}{LR_{i}\cdot\Pr\left(H_{1}\right)}\right)+\frac{\Pr\left(H_{2}\right)}{N_{2}}\sum_{i\in cat.2} \log_{2}\left(1+\frac{LR_{j}\cdot\Pr\left(H_{1}\right)}{\Pr\left(H_{2}\right)}\right)$

*Prior probabilities are rarely available for forensic experts, even though they can be inferred from several sources, such as witnesses, additional evidences or police investigations. As a consequence, one of the main advantages of this approach is that ECE can be automatically computed for the whole set of prior odds (i.e. the prior probability quotients), overcoming any lack of the forensic expert’s knowledge about the prior odds. In particular, in the so-called ECE plot (referred to previous section, Figures 1-2) three important components can be observed [2], as follows:*

1. *Observed curve (solid red) – it shows the ECE values calculated in accordance with the previous ECE equation for the LR values under evaluation;*
2. *Calibrated curve (dashed blue) – it represents the ECE values relevant to the LR values after the use of a pool adjacent violators (PAV) algorithm* [2,11,12]*. In particular, this procedure evaluates the unaltered discrimination power of the LR values set. As a consequence, the calibrated curve can be exploited to recognize the LR values showing the best performance in comparison with all the other LR sets showing the same discrimination power;*
3. *Null or reference curve (dotted black) – it describes the situation in which no value is assigned to the evidence. It is always the same and it can be used as a reference curve that shows the performance of a method always delivering LR=1.*

*The easiest way to interpret ECE plot when investigating the performance of a model is to evaluate the experimental set of LR values (represented by the observed curve – solid, red line), in comparison with the remaining calibrated (dashed blue) and null (dotted black) curves. In particular, the evidence evaluation process turns out misleading whenever the values of the observed curve are greater than the ones of the null curve. This means that the process of evidence evaluation introduces more misleading information than when it does not evaluate the evidence at all. In practice, the higher the observed curve, the worse the developed LR model as a lot of uncertainty is observed. For this reason, ECE plot was employed as a features selection technique too, in combination with the F-test approach. In fact, this approach allowed to remove from the database the variables providing an LR model with the observed curve exceeding the null one along the whole range of prior odds. Moreover, the amount of the residual uncertainty can be interpreted as an index of the discrimination power of the developed LR framework. In particular the point of the observed curve where log_10_(prior odds) = 0 represents the reduction of information loss resulting from the developed LR model for equal priors. Such point is named as $C_{llr}^{\exp}$ value. Furthermore, the point of the calibrated curve where the log_10_ of the prior odds is equal to zero is defined as $C_{llr}^{cal}$.*

*All the calculations and likelihood ratio modelling were performed with R software version 3.2.2 using scripts written by the authors and the Rcmdr package.*
