# Supplementary material for: Direct and indirect alcohol biomarkers data collected in hair samples - multivariate data analysis and likelihood ratio interpretation perspectives
Source: Data Brief. 2017 Mar 16;12:1–8. doi: 10.1016/j.dib.2017.03.026 (PMC5457474; doi:10.1016/j.dib.2017.03.026)
Supplement: Supplementary file 1 — Supplementary material [file mmc1.doc]

**Marina Ostankovitch, PhD	Date 16/01/2017**

Senior Scientific Editor

Data in Brief

Dear Dr. Ostankovitch,

we wish to confirm that there are no known conflicts of interest associated with this publication and there has been no significant financial support for this work that could have influenced its outcome.

We firmly confirm that the manuscript has been read and approved by all named authors and that there are no other persons who satisfied the criteria for authorship but are not listed. We further confirm that the order of authors listed in the manuscript has been approved by all of us.

We confirm that we have given due consideration to the protection of intellectual property associated with this work and that there are no impediments to publication, including the timing of publication, with respect to intellectual property. In so doing we confirm that we have followed the regulations of our institutions concerning intellectual property.

We further confirm that any aspect of the work covered in this manuscript that has involved either experimental animals or human patients has been conducted with the ethical approval of all relevant bodies and that such approvals are acknowledged within the manuscript (i.e. Ethical approval for the study was granted by the Ethical Committee of the Azienda Ospedaliero-Universitaria San Luigi Gonzaga of Orbassano (Protocol Number 0012756).

Yours sincerely,

Dott. Eugenio Alladio, PhD Student

PhD course in Chemical and Material Sciences

Università degli Studi di Torino – Department of Chemistry

Via Pietro Giuria 7 - 10125 Torino – Italy

Centro Regionale Antidoping e di Tossicologia “A. Bertinaria”

Regione Gonzole 10/1 – 10043 Orbassano (Torino) – Italy
